# miR-873a-5p Targets A20 to Facilitate Morphine Tolerance in Mice

**DOI:** 10.3389/fnins.2019.00347

**Published:** 2019-04-09

**Authors:** Jiangju Huang, Xia Liang, Jian Wang, Yan Kong, Zengli Zhang, Zhuofeng Ding, Zongbin Song, Qulian Guo, Wangyuan Zou

**Affiliations:** ^1^Department of Anesthesiology, Xiangya Hospital, Central South University, Changsha, China; ^2^National Clinical Research Center for Geriatric Disorders, Xiangya Hospital, Central South University, Changsha, China

**Keywords:** morphine tolerance, microRNA, miR-873a-5p, A20 (TNFAIP3), NF-κB

## Abstract

Long-term morphine administration leads to tolerance and a gradual reduction in analgesic potency. Noncoding microRNAs (miRNAs) modulate gene expression in a posttranscriptional manner, and their dysregulation causes various diseases. Emerging evidence suggests that miRNAs play a regulatory role in the development of morphine tolerance. In the present study, we hypothesized that miR-873a-5p is a key functional small RNA that participates in the development and maintenance of morphine tolerance through the regulation of A20 (tumor necrosis factor α-induced protein 3, TNFAIP3) in mice. We measured the percentage of maximum possible effect (MPE %) to evaluate the analgesic effect of morphine. The expression of miR-873a-5p and its target gene A20 were determined after the morphine-tolerant model was successfully established. Intrathecal injection with lentivirus to intervene in the expression of A20 and the miR-873a-5p antagomir was used to explore the role of miR-873a-5p in the development of morphine tolerance. Chronic morphine administration significantly increased the expression of miR-873a-5p, which was inversely correlated with decreased A20 expression in the spinal cord of morphine-tolerant mice. Downregulation of miR-873a-5p in the spinal cord attenuated and partly reversed the development of morphine tolerance accompanied by overexpression of A20. Similarly, A20 was upregulated by a recombinant lentivirus vector, which attenuated and reversed the pathology of morphine tolerance by inhibiting the activation of nuclear factor (NF)-κB. Collectively, our results indicated that miR-873a-5p targets A20 in the spinal cord to facilitate the development of morphine tolerance in mice. Downregulating the expression of miR-873a-5p may be a potential strategy to ameliorate morphine tolerance.

## Introduction

Morphine is often used to treat a variety of acute and chronic pain conditions (Bohn et al., 2000). However, the analgesic application of morphine is limited by undesirable effects such as morphine tolerance, drug dependence and respiratory depression (Harrison et al., 1998; Eriksen et al., 2006; Song et al., 2012). Morphine tolerance is defined as an attenuated analgesic effect, and an increasing morphine dosage is required to achieve adequate analgesia for long-term application (Song et al., 2010). Increasing evidence has shown that morphine tolerance is a complex process involving some cellular adaptations and molecular mechanisms (Arttamangkul et al., 2018). However, the underlying cellular and molecular mechanisms of morphine tolerance are not entirely understood.

MicroRNAs (miRNAs) are small noncoding RNA molecules that exert their function through posttranscriptional silencing of their target genes (Zhou et al., 2017) and may be potential therapeutic targets of many treatments for chronic pain (Kynast et al., 2013; Neudecker et al., 2016; Zhang et al., 2017). Increasing evidence suggests that miRNAs are the key modulators that accelerate morphine tolerance (Tapocik et al., 2016; Wang J. et al., 2016). In this study, we found that miR-873a-5p is unregulated in the spinal cord. Recent research found that miR-873 inhibits morphine-induced macrophage apoptosis, which may provide new molecular mechanisms for morphine addiction. IL-17 induces aberrant expression of miR-873, which targets A20 to regulate the production of inflammatory cytokines and chemokines and aggravate the pathological process of experimental autoimmune encephalomyelitis (EAE). However, the relationship between miR-873a-5p and morphine tolerance is unclear.

A20, also called tumor necrosis factor α-induced protein 3 (TNFAIP3), is one of the predicted target genes of miR-873a-5p (Liu X. et al., 2014; Li et al., 2015) and acts as a key regulator of inflammation and immunity by negatively regulating NF-κB signaling (Wertz et al., 2004). In this study, we tested the hypothesis that miR-873a-5p participates in the development and maintenance of morphine tolerance by targeting A20 in mice.

## Materials and Methods

### Animal Preparations

Specific pathogen-free (SPF) male ICR mice (24–28 g, 6–8 weeks old) were obtained from the Experimental Animal Service of Central South University. All animals were acclimatized for 3 days before the experiments started and were housed in plastic cages under a 12 h light/dark cycle. Four to five mice were housed per cage under pathogen-free conditions with soft bedding under a controlled temperature (22 ± 2°C) with *ad libitum* access to food and water. All procedures followed the guidelines approved by the Administration Committee of Experimental Animal Care and Use of Xiangya Hospital, Central South University. The study followed the ethical guidelines of the International Association for the Study of Pain (Zimmermann, 1983). All animals were divided randomly into different groups, and behavioral tests were performed and assessed by an independent observer blinded to treatment to minimize bias. All experiments were performed between 9:00 a.m. and 11:00 a.m.

### Morphine Tolerance Model and Behavioral Testing

To induce morphine tolerance, ICR mice received twice-daily injections of morphine [10 mg/kg, subcutaneous (s.c.)] for 7 consecutive days, and the control group was injected with the same volume of normal saline (Xu et al., 2017). Behavioral tests were performed before (basal response time) and 30 min after (drug response time) morphine was administered by tail-flick assay to assess morphine analgesia every 2 days. Briefly, the water temperature was adjusted to ensure a basal latency of 3–4 s, and a cut-off latency of 10 s was set to avoid tissue damage. Mice were placed in 52°C water to measure the latency of tail withdrawal (Fitting et al., 2016). The results were calculated as a percentage of maximum possible effect (%MPE), which was calculated by the following formula: MPE% = 100% × [(Drug response time – basal response time)/(cut-off time – basal response time)] (Harris and Pierson, 1964).

### Cell Culture

HT22 cells, an immortalized clonal mouse hippocampal cell line, were grown in high-glucose Dulbecco’s Modified Eagle’s Medium (Gibco) supplemented with 10% heated-inactivated horse serum (Gibco), 100 units/ml penicillin and 100 μg/ml streptomycin. The cells were maintained in an incubator with 5% CO_2._

### miRNA – Related Reagents

Antagomirs are chemically modified, cholesterol-conjugated, single-stranded RNA analogs (3-OMe-modified nucleotides) complementary to target miRNA. The miR-873a-5p antagomir (5′-AGGAGACUCACAAGUUCCUGU-3′), directed against murine miR-873a-5p, was purchased from GenePharma (Shanghai, China) as a negative control (5′-CAGUACUUUUGUGUAGUACAA-3′) to create an RNA sequence that is not encoded in the murine genome. Antagomirs were diluted to 20 μM.

### Construction of Lentiviral Vectors

Lentiviral vector plasmids encoding A20 were derived by cloning into the PgLV5/EF-1a/GFP vector, which expresses the GFP gene under control of the EF-1a promotor to increase A20 expression (LV-A20) (gene ID 21929), and the lentivirus negative control vector (LV-control) was purchased from GenePharma, China. The sequence CTACCTGAGTTCCTTCCCCTT was cloned into the pGLV3/H1/GFP vector to decrease A20 expression (LV-shA20), and the sequence TTCTCCGAACGTGTCACGT was the control (LV-NC). The vectors were cotransfected into HEK 293T cells using Polybrene (5 μg/ml). Lentiviral vector production, transfection and titration were performed according to protocols described previously (Szulc and Aebischer, 2008; Zou et al., 2011). The titer was 1 × 10^9^ TU/ml, and 5 μl of lentivirus was intrathecally injected into mice. The GFP fluorescence in the HT22 cells and spinal cord of mice was monitored to confirm successful transfection using a fluorescence microscope.

### Intrathecal Injection

The mice were held by the iliac crest to locate the spinous process of L6. A 30-gage, 0.5-inch needle attached to a 25 μl Hamilton syringe was inserted between the groove of the L5 and L6 vertebrae, and a tail flick indicated successful entry of the needle into the subdural space. When the tail flick was observed, antagomirs or lentivirus (5 μl) were slowly delivered into the intrathecal space with the other hand. Intrathecal injection did not affect baseline response time compared with the reaction time before injection.

### Western Blot

Spinal cord lumbar L4-L6 segment samples from mice in different groups were lysed in ice-cold RIPA buffer with protease inhibitors. Protein concentrations were measured with the BCA protein quantitative analysis kit (Biocolors, China). Proteins were separated via 10% sodium dodecyl sulfate polyacrylamide gel (SDS-PAGE) electrophoresis and transferred to polyvinylidene difluoride (PVDF) membranes. The membranes were blocked with 5% nonfat milk for 2 h at room temperature. After the membranes were blocked, they were incubated overnight with primary antibodies against A20 (mouse, 1:2000; Abcam), NF-κB (rabbit, 1:1000; Abcam), phosphorylated (p-)NF-κB (rabbit, 1:1000; Abcam), and β-tubulin (rabbit, 1: 2000; Abcam) at 4°C. The membranes were washed with TBST three times the next day and further incubated with goat anti-mouse or goat anti-rabbit IgG horseradish peroxidase-conjugated secondary antibody for 2 h at room temperature. The immune complexes were detected using super ECL Western blot detection reagents (Millipore, United States). The intensity of each band was determined using ImageJ software (National Institutes of Health, United States), and the expression of proteins was normalized to the expression of β-tubulin.

### Quantitative Real-Time PCR

Total RNA was extracted from the L4-L6 segments of the spinal cord using TranZol Up (TransGen Biotech, China). Reagents and kits were provided by GeneCopoeia (Guangzhou, China). All procedures were performed according to the manufacturer’s protocols. For quantification of mRNAs, total RNA (1 μg) from each sample was reverse-transcribed into cDNA with a random primer. The relative mRNA expression of A20 was normalized to the expression of the housekeeping gene GAPDH. Primer A20 forward: ATGGTGATGGAAACTGCCTCAT, reverse: ATTTCAGAGATTCCAGCTGCCA. GAPDH forward:AGGTCGGTGTGAACGGATTTG, reverse: TGTAGACCATGTAGTTGAGGTCA. For quantification of miRNAs, aliquots of total RNA (1 μg) from each sample were reverse-transcribed into cDNA. The miRNA primer sets for miR-873a-5p and U6 were purchased from GeneCopoeia (Guangzhou, China). RT-qPCR was performed using an all-in-One^TM^ miRNA RT-qPCR detection kit. The relative expression levels of miR-873a-5p were normalized to the expression of U6. The specificity of RT-qPCR primers was determined using a melt curve after amplification to show that only a single species of qPCR product was amplified from the reaction. The relative expression of A20 and miR-873a-5p was calculated by the 2^−ΔΔCt^ method. The RT-qPCR experiment was repeated 3 times.

### Immunofluorescence, Fluorescence *in situ* Hybridization, and Immunohistochemistry

Under deep anesthesia by intraperitoneal injection of pentobarbital sodium (10 mg/kg), mice were perfused transcardially with PBS (pH 7.4) followed by fresh 4% paraformaldehyde in 0.1 M PBS for 20 min. Then, L4-L6 lumber segments were removed, postfixed in the same fixative overnight and immersed in 30% sucrose in PBS. The next day, the spinal cords were rapidly frozen in liquid nitrogen and sectioned (10 μm). To detect the expression of A20, sections were preincubated in PBS containing 5% normal donkey serum and 0.3% Triton X-100 for 1 h. Double-staining immunofluorescence was used to determine the cell types in the spinal cord that express A20. The sections were incubated with the following antibodies: monoclonal anti-NeuN mouse (1:500, NOVUS, United States), monoclonal anti-GFAP mouse (1:600, Millipore, United States); monoclonal goat anti-Iba1 (1:400, Abcam, United States); monoclonal anti-GFP (1:200, Cell Signaling Technology); and rabbit anti-A20 antibody (1:200, Boster, China) at 4°C overnight. Sections were washed with PBS 3 times and then incubated with a secondary antibody labeled with Alexa Fluor-488 conjugated goat anti-rabbit (1:200, Jackson, United States) and Alexa Fluor-594 conjugated donkey anti-mouse or anti-goat (1:200, Jackson, United States) at room temperature for 2 h. Sections were washed 3 times with PBS, rinsed briefly in distilled water, dried and mounted with prolong Gold containing DAPI (Invitrogen, Carlsbad, CA, United States). Images were captured using a Leica DM5000B microscope equipped with a computer (Wang et al., 2017). A 5-DIG (digoxin)–and 3′-DIG-labeled mature miR-873a-5p miRCURY LNA detection probe (5′-DIG-AGG AGA CTC ACA AGT TCC TGC-DIG-3′) was synthesized by Exiqon (Denmark), and scrambled LNA probes were used as a negative control. Fluorescence *in situ* hybridization (FISH) was carried out using a FISH kit (Boster, China). Sections were treated with proteinase K for 15 min at 37°C and fixed in 4% paraformaldehyde for 10 min. Then, prehybridization was conducted with prehybridization buffer (50% formamide, 5 × SSC, 0.3 mg/ml yeast tRNA, 0.1 mg/ml heparin, 1 × Denhardt’s solution, 0.1% Tween-20, 5 mM EDTA in DEPC water) for 4 h at 54°C. The probe was diluted with hybridization buffer to 50 nmol and incubated for 16 h at 54°C. After hybridization, excess probe was removed by washing with SSC buffer. Sections were then incubated in Alexa Fluor 594-conjugated IgG fraction monoclonal mouse anti-digoxin. To identify the main protein coexpressed with miR-873a-5p, the sections used for FISH were further incubated overnight with primary antibodies, monoclonal rabbit anti-NeuN (1:400, Millipore, United States), monoclonal rabbit anti-GFAP (1:400, Millipore, United States), and monoclonal goat anti-Iba1 (1:400, Abcam, United States). Images were captured using a Leica DM5000B microscope equipped with a computer.

Spinal cords were fixed with fresh 4% paraformaldehyde and cut into 10-μm-thick sections. After heat-induced antigen retrieval, as mentioned before (Ding et al., 2017), sections were washed with 3% H_2_O_2_ for 10 min and blocked with 3% goat serum for 1 h. Then, the tissue sections were incubated with the miR-873a-5p miRCURY LNA detection probe overnight and incubated with goat anti-mouse IgG antibody for 20 min and streptavidin HRP for 20 min at 37°C the next day. Sections were measured using direct bonded aluminum (DAB) substrate (ZSGB-BIO, China). Images were captured using a Leica DM5000B microscope equipped with a computer. Image-Pro plus 6.0 was used to analyze the images.

### Luciferase Reporter Assays

The wild-type 3′-UTR of A20 mRNA (GUUCCUG) and mutant-type 3′-UTR of A20 mRNA (ACGUAAU) were inserted into pmiRGLO vectors (GenePharma, China). HEK 293T cells were cotransfected with miR-873a-5p mimics (5′-GCAGGAACUUGUGAGUCUCCU-3′) or miR-NC (5′-CUUUCCGAACGUGUCACGUTT-3′) and wild-type or mutant-type A20 3′-UTR. Cells were collected after transfection for 48 h, and luciferase intensity was detected with a dual luciferase reporter assay system as previously reported (He et al., 2010) (Promega Corporation, United States).

### Statistical Analysis

Mice were randomly assigned to treatment groups. Analyses were performed in a manner blinded to treatment assignments for all behavioral experiments. Data are expressed as the mean ± standard deviation (SD) and analyzed with GraphPad Prism software v7.0. Two-way ANOVA was applied to analyze repeated measurements from behavioral tests, followed by Bonferroni correction testing. One-way ANOVA with Bonferroni correction was applied to analyze immunofluorescence, Western blot and RT-qPCR data. No experimental data were missing or lost to statistical analysis. All statistical tests were two-tailed, and a *P* < 0.05 was considered statistically significant.

## Results

### miR-873a-5p Is Upregulated, While A20 Is Downregulated in the Spinal Cord of Morphine-Tolerant Mice

**FIGURE 1:**
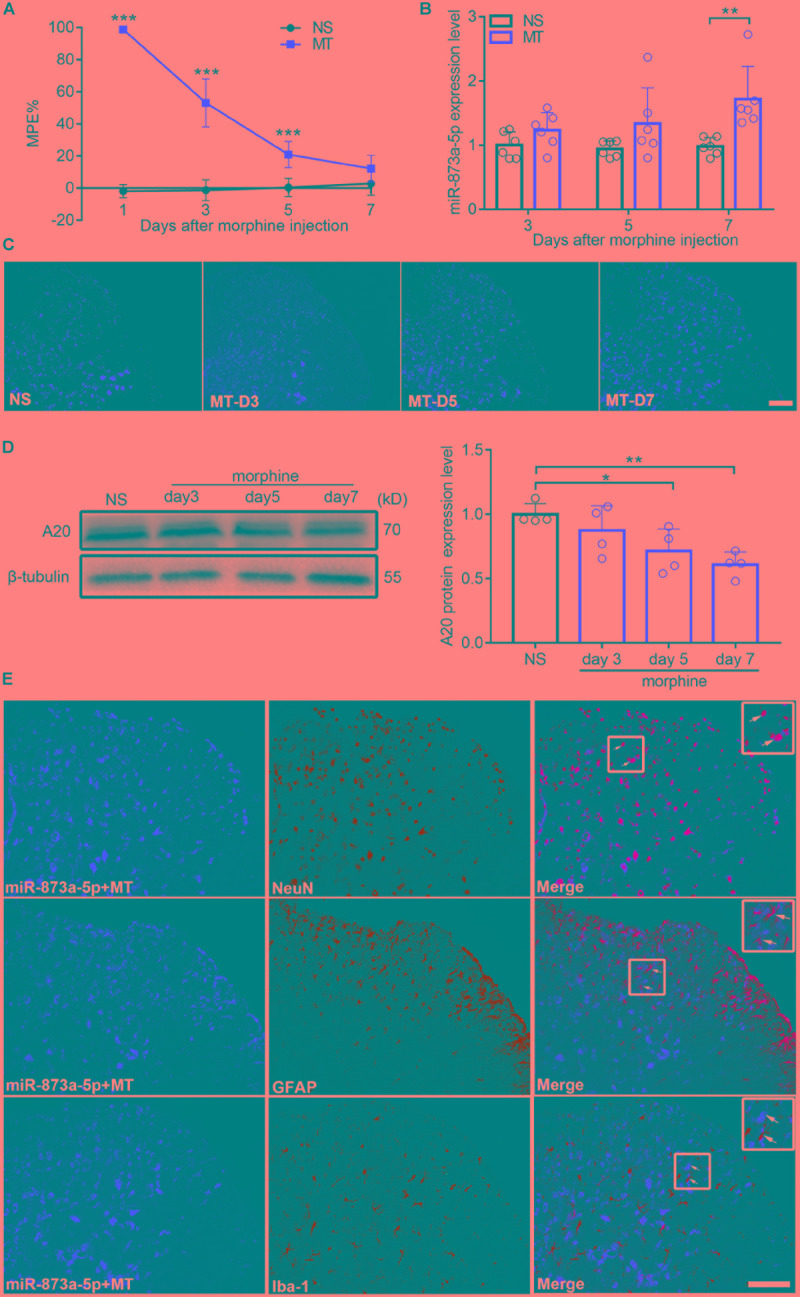
miR-873a-5p is upregulated, while A20 is downregulated in the spinal cord of morphine-tolerant (MT) mice. **(A)** Morphine-induced antinociception was assessed by the tail-flick test. Tail-flick latency was converted to MPE%. *n* = 8, ^∗∗∗^*P* < 0.001 compared with the NS group. **(B)** Real-time qPCR showed that miR-873a-5p expression increased in the morphine group 3, 5, and 7 days after chronic morphine administration, especially on day 7 compared to the expression in the control group. Data are expressed as the mean ± SD, *n* = 6 mice per group, ^∗∗^*P* < 0.01. **(C)** The staining of miR-873a-5p in the spinal cord, Scale bar = 100 μm. **(D)** Changes in the A20 protein expression level in the L4-L6 spinal cord were gradually decreased after the development of morphine tolerance, especially on day 7. *n* = 4 mice per group. Samples were collected on days 3, 5, and 7 following chronic morphine injection, ^∗^*P* < 0.05, ^∗∗^*P* < 0.01. **(E)** miR-873a-5p was assessed by *in situ* hybridization and staining of miR-873a-5p (red) with neurons (green, identified using NeuN), astrocytes (green, identified using GFAP) and microglia (green, identified using Iba1) of the spinal cord in morphine-tolerant mice. The data showed that miR-873a-5p was mainly expressed in neurons and astrocytes, whereas no expression was observed in microglia. Scale bar = 100 μm. Samples were collected on day 7 following chronic morphine injection.

After 7 consecutive days of s.c. morphine injection, the MPE% of mice from the chronic morphine treatment group (MT group) was significantly lower than that from the control group (NS group) (Figure 1A), indicating that the morphine tolerance model was successfully established. The L4-L6 of the spinal cord from mice were isolated, and real-time quantitative PCR and FISH were performed. Compared with the expression in the NS group, the expression of miR-873a-5p in the MT group began to increase on day 3 after morphine administration and was significantly higher on day 7 (NS vs. MT, *n* = 6, *P* < 0.05; Figure 1B,C), correlating with the development of morphine tolerance. To investigate the A20 protein expression level in the spinal cord of morphine-tolerant mice, Western blot analysis was conducted and showed that A20 expression was gradually decreased after morphine administration, especially after mice were administered morphine over 7 days (NS vs. MT, *n* = 4, *P* < 0.05; Figure 1D). To explore the localization of miR-873a-5p in the spinal cord of morphine-tolerant mice, miRNA *in situ* hybridization immunofluorescence was employed. Staining results revealed that the expression of miR-873a-5p was mainly located in neurons and astrocytes, and no expression was observed in microglia (Figure 1E).

### miR-873a-5p Directly Targets A20

**FIGURE 2:**
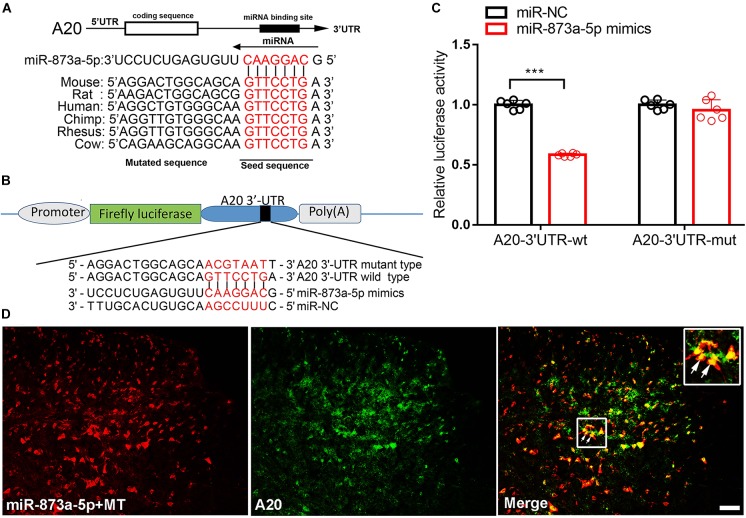
miR-873a-5p directly targets the A20 3′-UTR. **(A)** The 3′-UTR sequences of A20 containing the miR-873a-5p target regions and the binding sites of miR-873a-5p with the target sequence are well conserved among mammals. The binding site sequence is indicated in red bold letters. **(B)** Diagram of the seed sequence of miR-873a-5p matching the 3′-UTR of A20. Positions of the mutated nucleotides in miR-873a-5p and the 3′-UTR of A20. **(C)** The decreased luciferase activity induced by transfection with miR-873a-5p mimics was completely reversed by the mutant A20 3′-UTR vector. Data are expressed as the means ± SD, ^∗∗∗^*P* < 0.001, *n* = 6. **(D)** MiR-873a-5p costaining with A20 in the mouse spinal cord. Samples were collected on day 7 following chronic morphine injection, *n* = 3, scale bars = 100 μm.

To investigate the target of miR-873a-5p relevant to morphine tolerance, publicly available websites, including TargetScan and miRbase were searched. These databases showed that the 3′-UTR of the mouse A20 gene contains 7-mer (GTTCCTG) complementary bases to the seed region of miR-873a-5p and had a high degree of consistency among different species (Figure 2A). To support this conjecture, luciferase reporter assays were conducted. The 3′-UTR of the A20 mRNA fragment containing the wild-type (wt) or mutant-type (mut) binding site of miR-873a-5p was cloned into pmiRGLO vectors (Figure 2B). Luciferase reporter assay results showed that miR-873a-5p mimics markedly reduced the luciferase activity of the A20 3′-UTR-wt reporter vector compared with miR-NC, and the luciferase activity was reversed in the A20 3′-UTR-mut mimics group, demonstrating that A20 is a direct target gene of miR-873a-5p (*P* < 0.001; Figure 2C). FISH showed that miR-873a-5p was coexpressed with A20 in the spinal cord of mice (Figure 2D).

### The miR-873a-5p Antagomir Significantly Attenuates and Partly Reverses Morphine Tolerance in Mice

**FIGURE 3:**
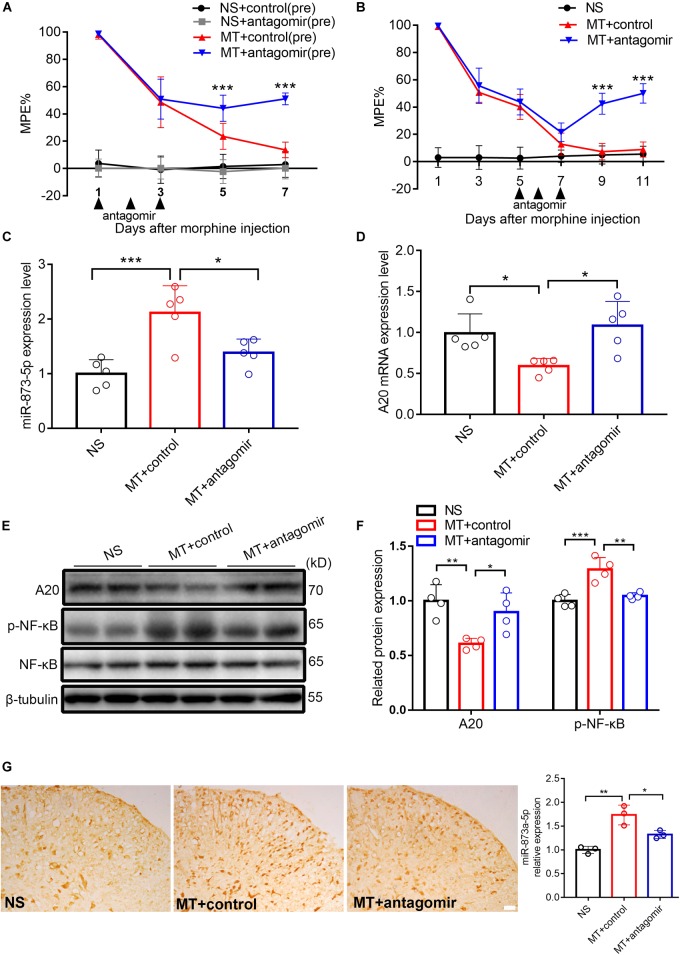
Downregulated miR-873a-5p significantly attenuates and partly reverses morphine tolerance in mice. **(A)** Preintrathecal injection of the miR-873a-5p antagomir for 3 consecutive days (from days 1 to 3 when morphine was injected) attenuated morphine-induced tolerance. *n* = 10, ^∗∗∗^*P* < 0.001 vs. the MT + control (pre) group. **(B)** Postintrathecal injection of the miR-873a-5p antagomir for 3 consecutive days (from days 5 to 7 after morphine injection) significantly reversed morphine-induced analgesic tolerance. *n* = 8, ^∗∗∗^*P* < 0.001 vs. the MT + control group. **(C)** The validation of miR-873a-5p antagomir transfection efficiency *in vivo* was tested by RT-qPCR. The MT + antagomir group had significantly lower miR-873a-5p expression than the MT + control group. *n* = 5, ^∗∗∗^*P* < 0.001 vs. the NS group; ^∗^*P* < 0.05 vs. the MT + control group. **(D)** RT-qPCR showing that miR-873a-5p antagomir administration reversed A20 mRNA expression in the spinal cord. **(E,F)** Western blot demonstrating that miR-873a-5p antagomir administration reversed A20 protein expression levels and downregulated p-NF-κB. *n* = 4, ^∗∗^*P* < 0.01, ^∗∗∗^*P* < 0.001 vs. the NS group; ^∗^*P* < 0.05, ^∗∗^*P* < 0.01 vs. the MT + control group. **(G)** *In situ* hybridization staining of miR-873a-5p in the spinal cord from the NS, MT + control and MT + antagomir groups. Images showing that miR-873a-5p expression was significantly downregulated in the mice treated with the antagomir; *n* = 3 mice per group, ^∗∗^*P* < 0.01, vs. the NS group; ^∗^*P* < 0.05 vs. the MT + control group, scale bars = 100 μm. Samples were collected 4 days after antagomir administration.

To investigate the role of miR-873a-5p in the development of morphine tolerance, a miR-873a-5p antagomir was used to downregulate the expression of miR-873a-5p in the spinal cord. The miR-873a-5p antagomir was intrathecally administered (20 μM, 5 μl) for 3 consecutive days from days 1 to 3 before the establishment of morphine tolerance. Behavioral results showed that there was no significant difference between the NS + control (pre) group and the NS + antagomir (pre) group, while the MT + antagomir (pre) group showed significant attenuation of the development of morphine tolerance from day 5 (MT + control (pre) vs. MT + antagomir (pre), *n* = 10, *P* < 0.001; Figure 3A), indicating that the miR-873a-5p antagomir attenuates the development of morphine tolerance.

To further investigate the contribution of miR-873a-5p to the maintenance of morphine tolerance, we posttreated mice with the miR-873a-5p antagomir from days 5 to 7 after morphine administration. Posttreatment with the miR-873a-5p antagomir partly reversed thermal hyperalgesia beginning at day 9 (MT + control vs. MT + antagomir, *n* = 8, *P* < 0.001; Figure 3B). The efficiency of the miR-873a-5p antagomir for downregulating miR-873a-5p was validated by qPCR. The miR-873a-5p antagomir reversed the overexpression of miR-873a-5p in the spinal cord of morphine-tolerant mice (MT + control vs. MT + antagomir, *n* = 5, *P* < 0.05; Figure 3C). To explore the effect of miR-873a-5p on morphine-induced spinal A20 expression, we collected the spinal cord and examined the expression changes in A20. Intrathecal injection of the miR-873a-5p antagomir significantly increased the expression of spinal A20 mRNA (MT + control vs. MT + antagomir, *n* = 5, *P* < 0.05; Figure 3D) and protein (MT + control vs. MT + antagomir, *n* = 4, *P* < 0.05; Figure 3E,F). Western blotting revealed that p-NF-κB expression was increased in the MT + control group (NS vs. MT + control, *n* = 4, *P* < 0.001; Figure 3F) but downregulated by intrathecal injection of antagomir in the MT + antagomir group (MT + control vs. MT + antagomir, *n* = 4, *P* < 0.01; Figure 3F). To explore the expression of miR-873a-5p in the spinal cord, miRNA *in situ* hybridization immunohistochemistry was performed. The *in situ* hybridization data showed that miR-873a-5p expression was significantly increased in the MT + control group compared with that in the NS group. Compared with the MT + control group, the MT + antagomir group showed significantly lower expression of miR-873a-5p in the mouse spinal cord (MT + control vs. MT + antagomir, *n* = 3, *P* < 0.05; Figure 3G). These data were consistent with previous RT-qPCR data and suggest that miR-873a-5p in the spinal cord partially reverses established morphine tolerance.

### Increased A20 Significantly Attenuates Morphine Tolerance by Inhibiting NF-κB in Mice

**FIGURE 4:**
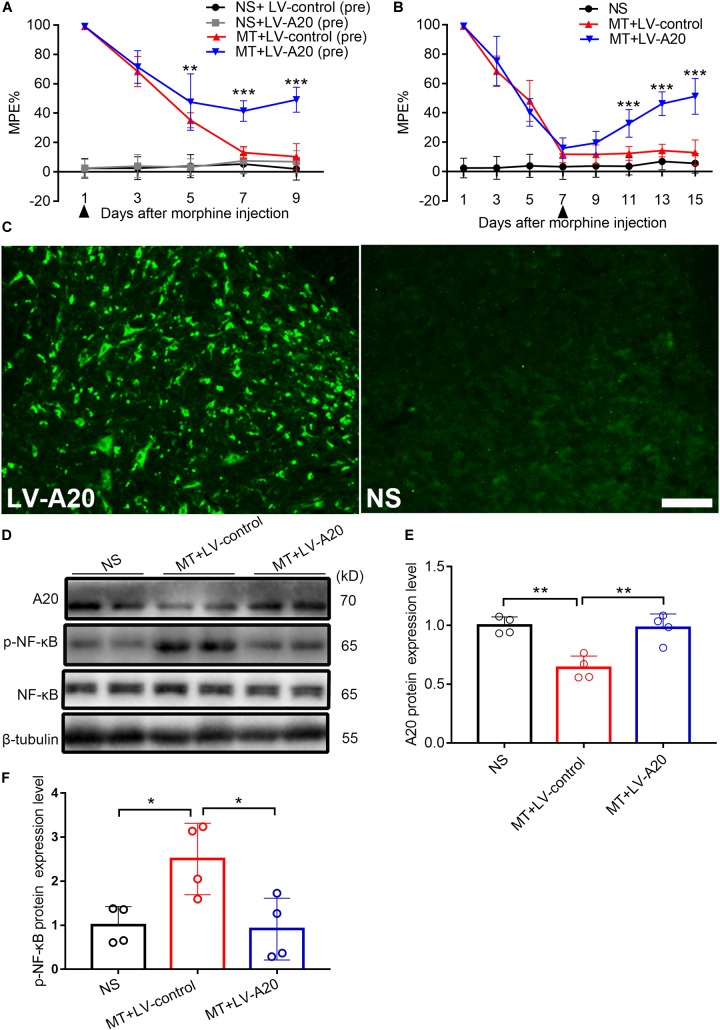
Overexpressed A20 prevents and reverses morphine tolerance in mice. **(A)** Mice were pretreated with the LV-control or LV-A20 vector before morphine tolerance was established. MT + LV-A20 (pre) treatment prevented the development of morphine tolerance compared with MT-LV-control (pre) treatment. *n* = 10,^∗∗∗^*P* < 0.001 compared with the MT + LV-control (pre) group. **(B)** Mice were injected with morphine twice a day for 15 consecutive days, and LV-control vector or LV-A20 vector was administered on day 7 after the establishment of morphine tolerance. MT + LV-A20 treatment attenuated morphine tolerance compared with MT + LV-control treatment. *n* = 10,^∗∗∗^*P* < 0.001 compared with the MT + LV-control group. **(C)** Image of enhanced green fluorescent immunofluorescence in the spinal cord after injection of LV-A20 or LV-control, indicating that the lentivirus was successfully transfected. *n* = 3, scale bars = 100 μm. **(D,E)** MT + LV-A20 upregulated A20 protein expression levels after lentiviral LV-A20 was injected. *n* = 4, ^∗∗^*P* < 0.01 compared with the NS group, ^∗∗^*P* < 0.01 compared with MT + LV-control. **(D,F)** p-NF-κB was significantly decreased when A20 was upregulated in the MT + LV-A20 group. *n* = 4, ^∗^*P* < 0.05 compared with the NS group, ^∗^*P* < 0.05 compared with MT + LV-control. Samples were collected 8 days after lentiviral vector administration.

To investigate the role of A20 in the pathology of morphine tolerance, we pretreated mice with LV-A20 or control lentiviral vector (LV-control), posttreated them with LV-A20 or LV-control and measured their behavioral response. Based on the behavioral data, treatment with morphine and LV-A20 (pre) significantly restored the antinociceptive effect of morphine beginning on day 5, especially on day 9 (MT + LV-control (pre) vs. MT + LV-A20 (pre), *n* = 10, *P* < 0.001; Figure 4A), and there was no significant difference between the NS + control (pre) group and NS + LV-A20 group. Based on the above results, LV-A20 caused no antinociception in the NS group, whereas there was significant antinociception in morphine-tolerant mice. After intrathecal administration of LV-A20 on day 7, we found that morphine tolerance was significantly reversed on day 15 (MT + LV-control vs. MT + LV-A20, *n* = 10, *P* < 0.001; Figure 4B). We collected the spinal cord and examined GFP expression. Green fluorescence was detected, suggesting that the lentivirus was successfully transfected into the cell body of the spinal cord (Figure 4C). Western blot data demonstrated that A20 was increased in the spinal cord of morphine-tolerant mice treated with LV-A20 (MT + LV-A20), while it was not found in the morphine-tolerant mice treated with LV-control (MT + LV-control) (MT + LV-control vs. MT + LV-A20, *n* = 4, *P* < 0.05; Figure 4D,E). The expression of p-NF-κB was significantly increased in the MT + LV-control group, while it was decreased in the MT + LV-A20 group by intrathecal injection with LV-A20 (MT + LV-control vs. MT + LV-A20, *n* = 4, *P* < 0.05; Figure 4D,F). These data suggested that overexpression of A20 could attenuate morphine tolerance by inhibiting NF-κB.

### A20 Is Responsible for miR-873a-5p-Mediated Morphine Tolerance in Mice

**FIGURE 5:**
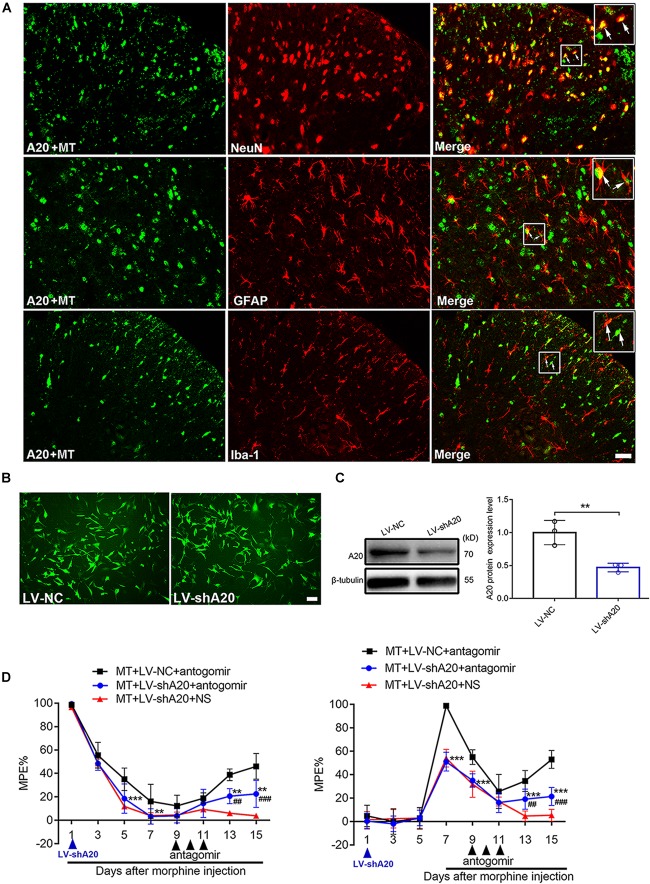
miR-873a-5p targets A20 to participate in morphine tolerance in mice. **(A)** Staining of the spinal cord for A20 (green), neurons using the NeuN antibody (red), astrocytes using the GFAP antibody (red), and microglia using the Iba1 antibody (red) in morphine-tolerant mice. A20 was mainly expressed in neurons and astrocytes and barely expressed in microglia. Scale bars = 100 μm. **(B)** GFP was detected in HT22 cells after transfection with lentivirus LV-shA20 and LV-NC (control); scale bars = 100 μm. **(C)** Western blot analysis showed that A20 protein expression was significantly decreased in HT22 cells after lentivirus (LV-shA20) transfection; *n* = 3, ^∗∗^*P* < 0.01 compared with the LV-NC group. **(D)** A20 is responsible for miR-873a-5p-mediated morphine tolerance in mice. LV-shA20 or LV-NC was intrathecally injected on day 1 with morphine injection, and the miR-873a-5p antagomir was intrathecally injected on day 9 when chronic morphine tolerance was established. MT + LV-shA20 or LV-NC was intrathecally injected 7 days before morphine was first injected, and the miR-873a-5p antagomir was intrathecally injected from days 9 to 11. *n* = 8, ^∗∗^*P* < 0.01, ^∗∗∗^*P* < 0.001 compared with the MT + LV-NC + antagomir group.^##^*P* < 0.01, ^###^*P* < 0.001 compared with the MT + LV-shA20 + NS group.

To determine the main cellular localization of A20 in the mouse spinal cord, double immunofluorescence staining was performed. Neurons, astrocytes and microglia were selected by staining for NeuN, GFAP and Iba1, respectively. A20 protein was mainly expressed in neurons and astrocytes (Figure 5A). To investigate the effect of LV-shA20 on the downregulation of A20, HT22 cells were transfected with LV-shA20 and GFP fluorescence was detected to confirm the success of lentivirus transfection (Figure 5B). The Western blot data showed that the expression of A20 in the LV-shA20 group was dramatically decreased compared with that in the LV-NC group (Figure 5C).

To further explore the functional relationship between miR-873a-5p and A20 in morphine tolerance, animals were treated with LV-shA20 to knockdown A20 before intrathecal injection with the miR-873a-5p antagomir in the morphine tolerance group (MT + antagomir + LV-shA20), and their behavior was then measured. Intrathecal injection of LV-shA20 (MT + LV-shA20 + antagomir) on the same day (Figure 5D, left) or 7 days before morphine injection (Figure 5D, right) downregulated A20 expression and significantly enhanced morphine tolerance compared with intrathecal injection of LV-NC (MT + LV-NC + antagomir), and morphine tolerance was alleviated by treatment with the miR-873a-5p antagomir compared with morphine tolerance in the group intrathecally injected with NS (MT + LV- shA20 + NS). These data suggest that downregulation of miR-873a-5p alleviates morphine tolerance by inhibiting spinal A20 in mice and that A20 is responsible for miR-873a-5p-mediated morphine tolerance.

## Discussion

Our study revealed that consecutive administration of morphine increased the expression of miR-873a-5p and decreased the expression of A20. To the best of our knowledge, this study is the first demonstration of a regulatory role for miR-873a-5p in the development of morphine tolerance through regulation of A20 expression ranging from molecular changes in cells to behavioral changes in animals.

Recently, more studies have indicated that small noncoding RNAs, miRNAs and lncRNAs are differentially expressed in the spinal cords of morphine-tolerant rats (Shao et al., 2018). Morphine alters the expression of miRNA, such as miR-219 (Wang et al., 2017) and miR-365 (Wang J. et al., 2016), and this disordered miRNA expression participates in chronic morphine-induced analgesic tolerance (Barbierato et al., 2015).

miR-873 was first identified in bovine alveolar macrophages (Xu et al., 2009). Recently, researchers found that miR-873 was expressed in various organs and implicated in various diseases. Inhibiting cardiomyocyte necrosis by suppressing receptor interacting protein kinase 1/3 (RIPK1/3) RNA synthesis and protein posttranslation decreases myocardial infarct size in an ischemia/reperfusion (I/R) injury animal model (Wang K. et al., 2016). miR-873 has been found to be decreased in breast tumors, negatively regulate the activity of estrogen receptor a (ERa) and decrease ERa phosphorylation, resulting in inhibition of cancer cell proliferation and growth (Cui et al., 2015). miR-873 has also been shown to increase lung adenocarcinoma cell proliferation and migration by targeting SRCIN1, and its expression is decreased in glioblastoma multiforme (GBM) tumor tissues and cell lines (Gao et al., 2015). However, previous studies have not shown an association of miR-873a-5p with morphine tolerance. Our results showed that miR-873a-5p expression was significantly increased in the spinal cord of morphine-tolerant mice and was mainly expressed in neurons and astrocytes. In addition, the analgesic effect of morphine was recovered by miR-873a-5p antagomir treatment. Our study exposed a new role of miR-873a-5p in regulating morphine tolerance.

As a central regulator of inflammation and widely inducible cytoplasmic protein, A20 is associated with several autoimmune disorders (Das et al., 2018). A20 is expressed in virtually all cell types, including nonhematopoietic cells (Kattah et al., 2018), dendritic cells (Zhang et al., 2016), neurons, astrocytes, and microglia (Voet et al., 2018). A20 has been reported as a rheumatoid arthritis susceptibility gene (Nititham et al., 2015), and reducing A20 expression in macrophages by myeloid-cell-specific deletion of the gene A20 in mice promptly aggravates rheumatoid arthritis by reducing lipopolysaccharide (LPS)-induced NLR family, pyrin domain-containing 3 (NLRP3) expression levels and negatively regulating NLRP3 inflammasome activation (Vande Walle et al., 2014). A20 levels were significantly reduced in leucine-rich repeat kinase 2 (LRRK)-mutated neurons, and this reduction involved the NF-κB pathway (Lopez de Maturana et al., 2016). Furthermore, A20 deletion in spinal cord astrocytes aggravated EAE by augmenting NF-κB activity (Wang et al., 2013). Lacking A20 specifically in microglia increased the numbers of microglia and critically controlled microglia activation as in conditions of neuroinflammation (Voet et al., 2018). Our results suggest that miR-873a-5p targets A20 to participate in morphine tolerance and is mainly expressed in neurons and astrocytes. The differences in the results observed across studies may be due to differences in the examined disease phenotypes, in the technical methods used and in the examined tissues.

As a classical negative immune regulatory protein, A20 limits the strength of NF-κB signaling (Garson et al., 1989). ABIN-1 (A20-binding inhibitor of nuclear factor κB) is an ubiquitin-binding protein. Recent reports found that the ABIN-1 protein interacts with the C-terminus of μ-opioid receptor (MOR) and inhibits MOR phosphorylation and internalization. The increase in NF-κB signaling by morphine was inhibited by the upregulation of ABIN-1 in the cytoplasm after chronic morphine exposure (Zhou et al., 2018). ABIN-1, which functions as an adaptor for A20, synergistically binds with A20 to restrict NF-κB signaling (Dziedzic et al., 2018). NF-κB regulates the biological processes of cell survival, inflammation, and other immune responses. NF-κB activation in the dorsal root ganglia and spinal cord contributes to the development of neuropathic pain (Tai et al., 2008; Liu M. et al., 2014). Liu X. et al. (2014) demonstrated that miR-873 induced by IL-17 stimulation promotes the production of inflammatory cytokines and aggravates the pathological process of EAE in mice through the A20/NF-κB pathway. The level of p-NF-κB expressed in neurons and astrocytes was increased in the rat spinal cord after establishment of the morphine tolerance model, and inhibition of NF-κB activation attenuated the development of chronic morphine-induced analgesic tolerance (Bai et al., 2014). Our results showed that p-NF-κB was significantly increased in the spinal cord of morphine-tolerant mice and was inhibited when A20 was upregulated by LV-A20.

**FIGURE 6:**
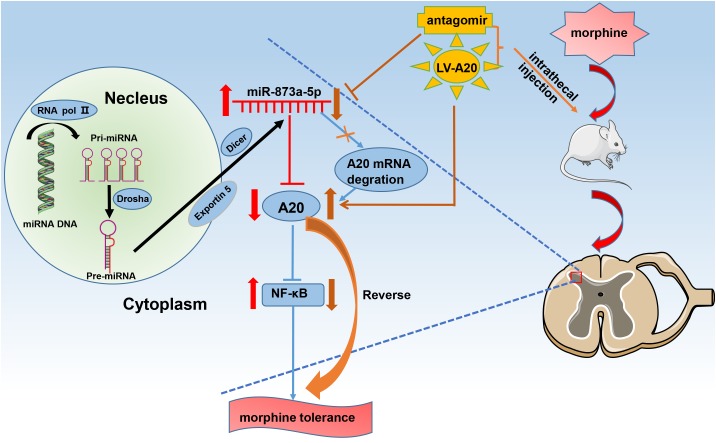
Schematic diagram for the proposed mechanism of morphine tolerance regulation by miR-873a-5p. Chronic morphine infusion sets up a cascade of events that leads to increased miR-873a-5p and decreased A20 expression, which ultimately causes morphine tolerance. The mice were intrathecally injected with the miR-873a-5p antagomir to inhibit mature miR-873a-5p or a lentiviral virus integrated into the host genome to increase the mRNA and protein expression of A20. This downregulation of miR-873a-5p or overexpression of A20 in the spinal cord of mice attenuated morphine tolerance. RNA pol II, RNA polymerase II; miR, microRNA.

Based on our data, we propose the following pathway. After chronic morphine administration, miR-873a-5p is upregulated, which facilitates translational repression of its target gene A20 and inhibits its expression in the neuron and astrocytes of the spinal cord. Downregulation of A20 expression is associated with NF-κB pathway activation, which contributes to the development and maintenance of morphine tolerance (Figure 6). The mice were intrathecally injected with the miR-873a-5p antagomir to inhibit mature miR-873a-5p or a lentiviral virus integrated into the host genome to increase the mRNA and protein expression of A20. This downregulation of miR-873a-5p or overexpression of A20 in the spinal cord of mice attenuated morphine tolerance.

This is the first study to demonstrate that miR-873a-5p prevents and revises morphine tolerance through A20. However, this study has several limitations. First, we chose only male ICR mice for our study, which is different from the general population and samples of clinical patients. Second, further experimental investigations, such as genetic deletion of miR-873a-5p in the spinal cord, will be needed to clarify the regulatory mechanisms of miR-873a-5p. Furthermore, clinical trials will be needed to test whether the findings of the study can be applied to humans.

## Conclusion

In conclusion, chronic morphine treatment increased the expression of miR-873a-5p through the A20/NF-κB pathway to facilitate morphine tolerance in mice. Intrathecal administration of the miR-873a-5p antagomir decreased miR-873a-5p expression, increased A20 protein levels and attenuated morphine tolerance. Our study expands our knowledge of the functional role of miR-873a-5p and provides a promising strategy for the treatment of morphine tolerance.

## Ethics Statement

This study was carried out in accordance with the recommendations of the ethical guidelines of the Administration Committee of Experimental Animal Care and Use of Xiangya Hospital, Central South University. The protocol was approved by the Administration Committee of Experimental Animal Care and Use of Xiangya Hospital, Central South University.

## Author Contributions

JH performed the experiments, collected and analyzed the data, and drafted the manuscript. XL performed the experiments and analyzed the data. JW, YK, ZZ, ZD, and ZS performed the experiments. WZ designed the study, analyzed the data and drafted the manuscript. WZ and QG revised the manuscript. All authors read and approved the final manuscript.

## Conflict of Interest Statement

The authors declare that the research was conducted in the absence of any commercial or financial relationships that could be construed as a potential conflict of interest.
